# Inattentional Blindness During Driving in Younger and Older Adults

**DOI:** 10.3389/fpsyg.2019.00880

**Published:** 2019-04-26

**Authors:** Raheleh Saryazdi, Katherine Bak, Jennifer L. Campos

**Affiliations:** ^1^Toronto Rehabilitation Institute, University Health Network, Toronto, ON, Canada; ^2^Department of Psychology, University of Toronto, Toronto, ON, Canada

**Keywords:** attention, aging, simulator, awareness, hazard, perceptual, cognitive, load

## Abstract

Age-related changes to perceptual and cognitive abilities have been implicated in an increased risk of collision in older adults. This may be due, in part, to their reduced ability to attend to potentially relevant aspects of their driving environment. An associated general phenomenon of inattentional blindness involves a failure to notice visually presented objects or events when attention is directed elsewhere. Previous studies of inattentional blindness using computer paradigms report higher incidence of this effect in older compared to younger adults. However, little is known about whether these age-related effects are observed during more complex, realistic, everyday tasks, such as driving. Therefore, the goal of this study was to explore whether younger and older adults differ in their awareness of objects in their driving environment when their attention is directed toward another primary driving task. This study took place in a high-fidelity, full field of view, driving simulator. Thirty-two younger (*M*_age_ = 25.41) and 32 older (*M*_age_ = 73.41) adults drove through 19 short scenarios and were asked to first judge whether their vehicle could fit between two rows of vehicles parked on either side of the road and then to perform the associated driving maneuver (i.e., drive through or drive around). On four critical trials, objects were placed on the side of the road that differed in terms of animacy. Specifically, animate objects consisted of 3D humans standing by a bus shelter and inanimate objects consisted of photographs of the same individuals on a bus shelter advertisement. Inattentional blindness was measured via a post-drive, tablet-based recognition task immediately following the critical trials. Results revealed high rates of inattentional blindness across both age groups, with significantly lower levels of awareness for inanimate objects compared to animate objects. Further, whereas younger adults demonstrated reduced inattentional blindness following the first critical trial, older adults did not show this immediate improvement in recognition performance. Overall, this study provides unique insights into the factors associated with age-related changes to attention and how they may affect important driving-related outcomes.

## Introduction

For many older adults, driving provides a sense of autonomy, contributes to community mobility, and helps to maintain overall quality of life. However, older adults are among the most vulnerable to traffic-related injuries and death caused by vehicle collisions (Transport Canada, 2014; Jackson and Cracknell, 2018). A recent systematic review by Vichitvanichphong et al. (2015) indicated that the most frequent driving errors made by older adults are those related to lane control, decision making, recognizing and responding to signs, visual scanning, and physical control of the vehicle. Older drivers are also particularly vulnerable to collisions during conditions of high sensory, perceptual, and cognitive load (e.g., when making left turns at intersections; Cantin et al., 2009; Road Safety Canada, 2011; Vichitvanichphong et al., 2015). These types of driving errors and increased collision rates are likely attributable to a variety of age-related changes, including but not limited to changes in sensory abilities (e.g., visual acuity, contrast sensitivity), perceptual abilities (e.g., time to contact estimation), and cognitive abilities (e.g., selective attention and working memory). Ultimately, the implications of these age-related effects on driving performance could include a reduced ability for older drivers to detect and/or interpret potential driving hazards, particularly when their perceptual and/or cognitive resources are taxed. Therefore, the goal of this study was to explore whether younger and older adults differ in terms of their awareness of objects in their driving environment when their attention is directed toward a primary driving task.

### Inattentional Blindness, Perceptual, and Cognitive Load During Driving

The failure to notice an object or event when attention is directed toward a primary task or target is referred to as “inattentional blindness” (Mack and Rock, 1998). In a classic study demonstrating this effect, observers who were shown a video of a basketball game and asked to count the number of ball passes, often failed to notice a gorilla that walked purposely across the basketball court (Simons and Chabris, 1999). The extent to which inattentional blindness is observed can depend on several factors including the primary task demands, the nature of the unexpected object/feature, and the characteristics of the observer themselves (Kreitz et al., 2016). In terms of individual characteristics, a number of studies have shown the rate of inattentional blindness to vary as a function of age. For example, Graham and Burke (2011) replicated the Simons and Chabris (1999) study with younger and older adults and revealed that older adults were even more susceptible than younger adults to inattentional blindness in this task (i.e., much less likely to notice the gorilla). Other studies have replicated this increased susceptibility of older adults to exhibit inattentional blindness using a variety of computer-based paradigms (e.g., Stothart et al., 2015, 2016; Horwood and Beanland, 2016). Very little, however, has been explored with regards to whether age-related differences in inattentional blindness are also observed during complex and realistic everyday tasks such as driving.

During multisensory, multitasking activities such as driving, the ability to attend to objects in the environment that are not immediately relevant to the task itself can be particularly challenging. As such, broad object awareness may generally be limited during driving compared to less complex tasks, particularly during conditions of higher cognitive and perceptual load. For instance, cognitive load can be increased during driving by the introduction of multitasking requirements (e.g., listening/talking, holding information in memory, navigating; Strayer and Johnston, 2001; Strayer et al., 2003, 2013; Horrey and Wickens, 2006; Blalock et al., 2014; Cuenen et al., 2015; Donmez and Liu, 2015; Ebnali et al., 2016; Svetina, 2016; Murphy and Greene, 2017a; Caird et al., 2018; Wechsler et al., 2018) and perceptual load may be introduced by, for example, environmental clutter (e.g., traffic, buildings, signs, pedestrians; Marciano and Yeshurun, 2012, 2015; Stinchcombe and Gagnon, 2013; Ericson et al., 2017; Michaels et al., 2017), or by increasing perceptual task difficulty (e.g., judging maneuverability around closely arranged obstacles; Murphy and Greene, 2015, 2016). Previous studies with younger drivers have demonstrated more instances of inattentional blindness during conditions of higher compared to lower cognitive and perceptual load (e.g., Most and Astur, 2007; Blalock et al., 2014; Murphy and Greene, 2015, 2016, 2017a,b; Ericson et al., 2017; see Murphy et al., 2016 for a review). For instance, Murphy and Greene (2015, 2016) investigated the effects of perceptual load on inattentional blindness by asking drivers to make perceptual gap judgements about whether their car could fit between a row of parked cars while manipulating perceptual difficulty (i.e., clearly too wide/narrow vs. closely approximating the width of the driver’s vehicle). Their results demonstrated greater rates of inattentional blindness to roadside objects during the higher load conditions compared to the lower load conditions.

Importantly, very little is understood about how perceptual load affects inattentional blindness in older adults. Because there are well-documented age-related changes to, for instance, attentional capacity (Craik and McDowd, 1987; McDowd and Craik, 1988) and inhibitory control of attention (Hasher and Zacks, 1988; Lustig et al., 2007), it may be expected that older adults would demonstrate differences in inattentional blindness under load compared to younger adults (Graham and Burke, 2011). For instance, the attentional capacity model of cognitive aging posits that older adults have a more limited attentional capacity than do younger adults (Craik and McDowd, 1987; McDowd and Craik, 1988). As such, older adults might be less likely to detect an object that is not relevant to the primary driving task and hence may be *more* susceptible to inattentional blindness (e.g., Graham and Burke, 2011; Horwood and Beanland, 2016). Other theories of age-related changes in attention posit that older adults are less able to inhibit their awareness of information that is irrelevant to their primary task (Hasher and Zacks, 1988), suggesting that they may have increased awareness of environmental objects/features and thus, may be *less* susceptible to inattentional blindness. Although past studies of inattentional blindness provide support for the predictions made by the attentional capacity model (e.g., Graham and Burke, 2011; Horwood and Beanland, 2016; Liu, 2018), less is understood about the role of perceptual/cognitive load on these effects, or the role of different object characteristics. It is possible, for instance, that under different primary task loads, when using different measures of awareness, and/or with different degrees of object relevance, these age-related differences in inattentional blindness may vary (Michaels et al., 2017). Driving experience is another important consideration as older adults typically have accumulated more years of driving than younger adults, which in turn could compensate for their age-related functional declines. However, previous studies examining years of driving experience have revealed little to no effect on various driving measures (e.g., Shinar et al., 2005; Kass et al., 2007; Smahel et al., 2008) and very little is understood about the effects of lifetime driving experience on attention during driving.

### Effects of Object Type: Role of Animacy

Objects that are more salient and/or more relevant to the primary task may receive greater levels of awareness. One object feature that has been shown previously to affect rates of inattentional blindness is animacy. Specifically, past studies using simple computer-based tasks with static stimuli have reported lower rates of inattentional blindness for animate (e.g., animals/humans) compared to inanimate stimuli (e.g., tools/transportation vehicles; Calvillo and Jackson, 2014; Calvillo and Hawkins, 2016). In the context of driving, the characteristic of animacy is particularly important because it determines whether the object could, at any moment, become relevant to the primary driving task (i.e., the need to initiate a reactive response to things that can move). A driver should be prepared to avoid an animate object that has the potential to enter the roadway, whereas a stable, inanimate roadside object would be less of a concern. A study by Pammer et al. (2015), in which participants were presented with photographs of driving scenes, revealed a reduction in the rate of inattentional blindness as the threat of a hazard increased (e.g., a child on the side of the road compared to an adult). What is not clear is whether these effects would be observed during dynamic driving tasks, and/or under conditions of higher load. Assuming there are limited attentional resources, the awareness of some objects (e.g., animate) may be prioritized over others. However, it is also possible that once the driving load (perceptual and/or cognitive load) becomes too great, the effects of animacy are diminished. What is also not yet known is whether older adults’ awareness is differentially affected by animacy compared to younger adults’. For instance, age-related reductions of inhibitory control could be advantageous when an object is potentially relevant (animate) and leads to the detection of a hazard to be avoided, whereas it could be disadvantageous if the object is irrelevant (inanimate) and directs attention away from the primary task of driving. Therefore, the objectives of the current study were to evaluate inattentional blindness in younger and older adults, both in terms of animate and inanimate roadside objects during an active driving simulator task. Specifically, the animate objects consisted of 3D humans standing by a bus shelter and the inanimate objects were photographs of the same individuals on the bus shelter advertisement. To introduce load during driving, a gap judgment task (similar to Murphy and Greene, 2015, 2016) was implemented whereby the participants’ primary task was to determine whether they could drive between two rows of parked vehicles or whether they had to drive around (and then execute the associated maneuver). The primary goal of the gap judgment task was to introduce a sufficiently attention-demanding secondary task and was not intended as a manipulation to evaluate the specific effects of high versus low perceptual and/or cognitive load. The rate of inattentional blindness was measured via a post-drive, tablet-based recognition task immediately following the critical trials.

## Materials and Methods

### Participants

Seventy-one participants were recruited through advertisements posted in the local Toronto community. Due to simulator sickness, seven of the participants (5 older adults and 2 younger adults) were not able to complete the experimental task and were therefore excluded from the study. The final sample included 32 healthy younger adults (Age range = 20–35, *M* = 25.41, *SD* = 4.58, Male = 16) and 32 healthy older adults (Age range = 65–90, *M* = 73.41, *SD* = 6.19, Male = 18). All participants completed a pre-screening questionnaire to ensure that they met the eligibility criteria, namely age (younger adults 20–35; older adults 65+), and having a valid driver’s license, 2 years of recent driving experience, normal or corrected-to-normal visual acuity (verified with in person screening – see below), and no history of serious physical, neurological, or psychological disorders. Individuals who were eligible were invited to participate in the experimental session and were compensated $10 per hour for their participation. The protocol for the present study was approved by the University Health Network’s Research Ethics Board (REB 17-5596).

### Demographics, Sensory, and Cognitive Measures

Participants were administered a series of assessments in person, including a health history and demographics questionnaire, driving habits questionnaire (Owsley et al., 1999), and motion sickness susceptibility questionnaire (Golding, 2006). Visual acuity was assessed using the Early Treatment Diabetic Retinopathy Study visual distance test (ETDRS; Ferris et al., 1982) and −0.2 to 0.5 logMar units was considered as the acceptable range for the normal to near-normal visual acuity cut-off (International Council of Ophthalmology, 2002). In order to characterize the cognitive abilities of younger and older adults, a series of standardized cognitive tests were administered. The WAIS-III forward and backward digit span (Wechsler, 1997) was administered as a measure of working memory, with lower scores indicating poorer performance. For all of the remaining cognitive measures described below, a lower score indicates better performance. The Stroop test (Stroop, 1935) was used as a measure of inhibition and was scored by subtracting the number of correct words uttered per second in the neutral condition (colored asterisks) from the incongruent condition (word-color match/mismatch). The Trail Making Tests A and B (Reitan, 1955) were used as a measure of executive function with the score calculated as the completion time difference between the two versions (B minus A). In addition, we administered the Useful Field of View Test (UFoV; Ball and Owsley, 1993), a computerized task in which participants must identify a central object and the location of a peripheral object in the presence/absence of distractors. This task computes sub-scores for selective attention, divided attention, processing speed, as well as a total composite score, and is considered to be a strong predictor of driving collision frequency in older adults (Ball and Owsley, 1993). Finally, all older adults were administered the Montreal Cognitive Assessment (MoCA; Nasreddine et al., 2005) to screen for mild cognitive impairment. Due to technical error, we were not able to compute scores for one older adult for the digit span, three younger adults for the Stroop, and one younger adult for Trails A and B.

### Stimuli and Apparatus

#### Driving Simulator

**FIGURE 1:**
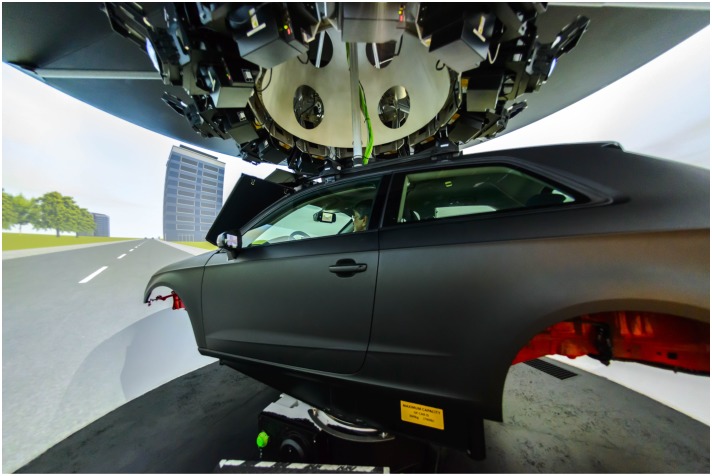
DriverLab at the Toronto Rehabilitation Institute – University Health Network (written informed consent was obtained from the depicted individuals for the publication of this image).

The study took place at the Toronto Rehabilitation Institute’s Challenging Environment Assessment Laboratory and used DriverLab, a state-of-the-art driving simulator (Figure 1). DriverLab is equipped with a full-sized passenger vehicle (Audi A3) containing all of its original internal components (e.g., steering wheel, gas/brake pedals, seats, and dashboards). The vehicle is completely surrounded by a 360-degree field of view visual projection system (12 Eyevis ESP-LWXT-2120, 1920 × 1200; 120 Hz projectors) and has vehicle-integrated surround sound (Pioneer VSX-45 Receiver, 5.1 sound; JL Audio powered sub and Focal speakers).

#### Driving Scene/Scenario

**FIGURE 2:**
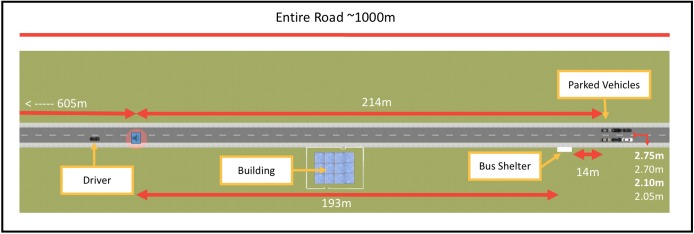
A top-down view of the driving scenario.

The driving scenes/scenarios were developed and presented using Oktal SCANeR Studio version 1.7 and MATLAB R2015b (The MathWorks Inc., 2015). The driving scenarios consisted of a straight rural road with no active traffic (see Figure 2). The number of objects in the scenarios (e.g., buildings and trees) was kept minimal and was balanced on both sides of the road. The entire road was approximately 1,000 m long. At approximately 820 m from the start of the drive, two rows of three vehicles were parked on either side of the road. The range of distances between the two rows of parked vehicles was 2.05–2.75 m apart. A bus shelter was positioned 14 m before the rows of parked vehicles on the right hand side of the road.

#### Target and Distractor Stimuli

**FIGURE 3:**
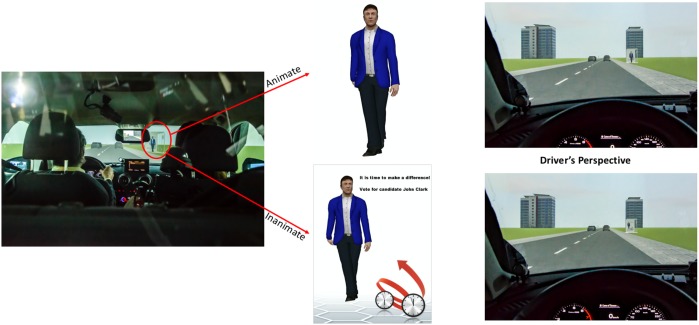
Example stimuli including animate **(top)** and inanimate **(bottom)** objects (written informed consent was obtained from the depicted individuals for the publication of this image).

The target objects within the driving scene and the target and distracter objects that were presented via the tablet during the post-drive recognition task were created using Google SketchUp, version 17.2.2 and the Google 3D warehouse. Target objects presented during critical driving trials were either animate or inanimate. Animacy was manipulated by presenting either a 3D person standing in the bus shelter (animate), or the same person depicted on a full height advertisement in the bus shelter (inanimate). Specifically, we included four people for the critical trials (2 males, 2 females depicted as either animate or inanimate) and four different people in filler trials (2 males, 2 females depicted as either animate or inanimate). In order to control for other non-animacy related differences between the two different animacy trial types, the advertisement content present in the inanimate trials was also replicated within the bus shelter during animate trials (i.e., in the animate trials, the same advertisement without the person was positioned directly behind the 3D person). This resulted in manipulating animacy while controlling for the general visual content in both the animate and inanimate trials (see Figure 3). Note that although the size of the person in the inanimate is smaller than the animate person, it is still quite large and clearly visible (e.g., the height of the inanimate man in the suit on Figure 3 is 1.5 m).

#### Tablet-Based Response Measures

Additional sets of 3D images were obtained and converted to 2D graphics for the four trials involving the tablet test of inattentional blindness. Importantly, each of these trials included two human characters; one who was present in the driving scene (critical target) and one who was not (competitor), as well as two plausible, non-human roadside objects (e.g., bicycle, mailbox, and newspaper stand), none of which ever appeared in the driving scene. These four images were presented on a 10″ Samsung Galaxy tablet. Each image was depicted on a white background at a 220 × 260 pixel resolution and the image location of each object type (within the four quadrants) was randomized across trials. Although the critical target and the human competitor for each trial matched in terms of their sex, they differed in terms of other characteristics (e.g., posture, clothing, and hairstyle), which provided additional unique identifiers apart from just different facial features across targets/competitors. This is an important detail, given that previous literature has suggested that face stimuli are unique in that they are processed to a greater degree than non-face stimuli under higher load conditions (Lavie et al., 2003).

## Procedure

After providing informed written consent, participants were asked to complete the set of questionnaires mentioned earlier. They were then guided to DriverLab and were assisted in adjusting their seat and getting familiarized with the vehicle. During the entire driving session, which lasted approximately 30 min, one researcher always sat in the passenger seat of the car with the participant, and another researcher monitored the experiment from outside the simulator.

### Familiarization Phase

Participants were first required to complete a 5 min familiarization phase, which involved driving along a straight rural road that was similar in nature, but not identical to the main experimental scenarios. During this phase, participants were asked to maintain a speed of 80 km/h (∼50 miles/h), make several lane changes, and drive on the shoulder. Participants were instructed to obey all traffic rules as they completed the driving task (e.g., obey speed limits, use their indicator before changing lanes, and avoid obstacles). Upon completion of the familiarization phase, participants were asked to report any symptoms of motion sickness and confirm that they were comfortable with proceeding to the experimental phase.

### Experimental Phase

Participants were instructed to drive along a straight, one-way rural road in a series of short driving trials. They started from a parked position on the road and drove straight forward within the right-hand lane. It was explained to them that they would come across a section of the road with vehicles parked on either side of the road and, upon approaching these vehicles, they would have to make a gap judgment to determine whether they could fit between the vehicles or whether they would need to navigate around the vehicles by driving on the shoulder. They were assured that the driving simulator car’s physics had been turned off so they would not feel any physical impact if they made an error in the gap judgment. Gap values were either “plausible” to drive through (Wide: 2.75 m, 2.70 m) or “implausible” to drive through (Narrow: 2.10 m, 2.05 m) with respect to “fit-ability”. The width of the driver’s vehicle was 1.8 m and although physically they could drive through the narrow gap, it would have been difficult and perceived as potentially “dangerous” to do so.

At a defined decision point before reaching the parked vehicles (marked by an auditory tone, see red circle and sound icon on Figure 2), participants were instructed to signal left if they believed that they could drive through the parked vehicles and signal right if they believed that they had to drive around the parked vehicles. Importantly, they were asked to follow their signal by performing the associated driving maneuver. Participants were also told to maintain the same speed as during the practice phase (80 km/h) and to bring the car to a stop at their own comfortable pace after driving through/around the parked vehicles. After confirming that participants understood the instructions, they were informed that they could not converse with the experimenter while driving.

The experimental phase involved 19 short driving scenarios (2 practice + 16 experimental + 1 probe trial). The four different gap sizes were equally represented across the 16 experimental trials and the two practice trials included one narrow (2.05 m) and one wide gap (2.75 m). Across the 16 experimental trials there were also four instances of each of the following object conditions: bus shelter with an animate object (3D person), bus shelter with an inanimate object (advertisement), an empty bus shelter, and no bus shelter. The two practice trials had empty bus shelters. Across the experimental trials, these object conditions were equally divided among the gap conditions. The pairing of conditions was accomplished using a list design, varying whether a wide or narrow gap size accompanied an animate or an inanimate roadside object. Although all participants were presented with all four combinations across the four critical trials, they were only presented with a particular object once. For example, each participant would be presented with the man in the suit depicted in Figure 3 only in one of the four critical trial combinations: (1) animate and narrower gap; (2) inanimate and narrower gap; (3) animate and wider gap; (4) inanimate and wider gap. The same character was associated with the same critical trial across participants (e.g., the man in the suit in Figure 3 was always the first critical trial). Moreover, to ensure that the participants could, in fact, perceive the roadside object when their attention was not divided by the gap judgment task, we also included a probe trial at the end of all experimental trials, in which participants were presented with a bus shelter that contained an object (an animate or inanimate man) but they did not have to make a gap judgment (i.e., there were no parked vehicles on the road). All participants did in fact see the roadside object (performance was at 100% for both animate and inanimate probe trials) and thus no further exclusions were required.

On four of the 16 experimental trials, inattentional blindness was assessed using a forced-choice recognition task presented on a tablet immediately after the driving trial. Specifically, participants were asked to select an image of the object that they recognized from the preceding trial. Each trial was coded for accuracy (correct/incorrect) and incorrect trials were further coded for same category error (choosing the human competitor) versus different category error (choosing a non-human object). Upon the completion of the experimental task, participants were asked to rate their level of simulator sickness on a scale of 0 (no sickness) to 20 (extreme sickness, Keshavarz and Hecht, 2011). Both younger and older adults reported low and similar rates of sickness (*M* = 2.31, *M* = 2.02, respectively). Finally, participants were asked to complete the remaining set of cognitive performance measures. Overall, the study took approximately 1.5–2 h to complete. Considering the possibility that time of the day could differentially affect younger and older adults’ performance (Anderson et al., 2014), we balanced the time of testing for each age group by having approximately the same number of younger and older adults tested in the morning and afternoon sessions.

### Data Analyses

All statistical analyses were conducted using R Version 3.3.3 (R Core Team, 2017). The comparisons of younger and older adults’ performance on baseline measures were analyzed with independent samples *t*-tests (see Table 1). All primary experimental dependent measures were analyzed using logistic mixed-effects analyses. These analyses were carried out using the lme4 package Version 1.1-15 (Bates et al., 2015) and lmerTest package Version 3.0-1 (Kuznetsova et al., 2017). Age (younger vs. older) was treated as a between-participants factor, and gap size (wide vs. narrow) and animacy (animate vs. inanimate) were treated as within-participant factors. The dependent measures were “accuracy” in gap judgment (accuracy here reflects driving *through* the parked vehicles when the gap was clearly wide enough and driving *around* the parked vehicles when the gap was too narrow to be considered safe to drive through) and accuracy in the rate of detection of the animate/inanimate object, which were both treated as binary outcomes (correct/incorrect). The random effects structure included a random intercept term for participant, a by-participant slope term for gap size in the analysis of gap judgment accuracy, and a by-participant slope term for animacy in the analysis of inattentional blindness. The results of these analyses are presented in Table 2.

**Table 1:** Participant demographics and baseline measures.

	Younger adults (*N* = 32)	Older adults (*N* = 32)	
	*M (SD)*	*M (SD)*	*p*-value
**Demographics**			
Age (years)	25.41 (4.58)	73.41 (6.19)	<0.001^∗^
Education (years)	17.19 (2.28)	18.25 (3.44)	0.151
**Vision**			
ETDRS left eye^1^	0.06 (0.18)	0.19 (0.16)	0.003^∗^
ETDRS right eye^1^	0.03 (0.17)	0.18 (0.14)	<0.001^∗^
**Cognition**			
MoCA^2^	–	26.09 (2.99)	–
Digit span^3^	18.13 (3.23)	16.61 (3.21)	0.067
Stroop^4^	0.56 (0.19)	0.47 (0.18)	0.077
Trails^5^	27.74 (9.97)	62.19 (52.07)	<0.001^∗^
UFoV^6^			
Processing speed	17.41 (4.72)	21.91 (20.24)	0.229
Divided attention	18.41 (7.46)	70.03 (87.10)	0.002^∗^
Selective attention	42.41 (23.78)	171.72 (96.49)	<0.001^∗^
Total score	78.22 (29.22)	263.66 (164.22)	<0.001^∗^

*^∗^Significance level of p < 0.05 when comparing younger and older participant group scores; ^1^Early Treatment Diabetic Retinopathy study scores in logMAR units; ^2^Montreal Cognitive Assessment, adjusted for years of education; ^3^score out of 30; ^4^number of correct words per second from neutral condition to incongruent condition; ^5^Trails B-A; ^6^Useful Field of View.*

**Table 2:** Summary of the results for mixed effect analyses.

Effect	Estimate	*SE*	*Z*	*p*
**Gap judgment accuracy**				
(Intercept)	2.43	0.23	10.78	<0.001
Gap size	−0.23	0.29	−0.79	0.432
Age	0.38	0.19	2.06	0.039
Gap size × age	−0.47	0.26	−1.82	0.069
**Inattentional blindness**				
(Intercept)	0.59	0.25	2.36	0.018
Age	0.35	0.23	1.56	0.119
Animacy	0.83	0.21	3.99	<0.001
Age × animacy	0.29	0.18	1.55	0.121
**Inattentional blindness growth curve analysis**				
(Intercept)	0.75	0.25	3.00	0.003
Linear	−0.15	0.38	−0.38	0.702
Quadratic	0.52	0.42	1.24	0.216
Age	0.43	0.25	1.72	0.086
Animacy	0.95	0.21	4.58	<0.001
Linear × age	0.78	0.38	2.04	0.042
Quadratic × age	0.68	0.42	1.62	0.105
Linear × animacy	0.28	0.38	0.74	0.460
Quadratic × animacy	−0.30	0.50	−0.61	0.545
Age × animacy	0.30	0.21	1.47	0.142
Linear × age × animacy	0.12	0.38	0.31	0.759
Quadratic × age × animacy	0.21	0.50	0.43	0.669

*Contrast coding: age (younger adults = 1, older adults = −1); perceptual gap judgment (narrow gap = 1, wide gap = −1); animacy (animate = 1, inanimate = −1).*

## Results

### Demographic, Sensory and Cognitive Measures

Both younger and older participants were similar in terms of demographic background, with most having completed, or were in the process of completing a university-level degree. To compare the driving habits of younger and older adult participants, we compiled an average score from the information collected on the driving habits questionnaire, accounting for both the average number of trips driven and average distances traveled on a weekly basis. The two age groups were very similar in terms of the average kilometers driven per week (Younger Adults: *M* = 142.31 km, *SD* = 186.79; Older Adults: *M* = 131.48 km, *SD* = 142.72). Younger and older adults also did not differ in terms of their susceptibility to motion sickness.

Younger and older adults were also compared for each of the measures of sensory and cognitive functioning. Whereas younger adults had better visual acuity than older adults overall, both groups’ average score of left and right eye acuity fell within the normal to near-normal range (−0.2 to 0.5 logMar units; International Council of Ophthalmology, 2002). In terms of the battery of cognitive measures, there were no significant group differences on the digit span test (*p* = 0.067) or the Stroop test (*p* = 0.077). However, younger adults performed significantly better than older adults on the Trail Making (*p* < 0.001) and the UFoV (*p* < 0.001) tests. Notably, the age-related differences in UFoV were evident for measures of divided attention (*p* = 0.002) and selective attention (*p* < 0.001), but not processing speed (*p* = 0.229).

Nine older adults scored below the MoCA cut-off for mild cognitive impairment (<26), however, these participants were still included in the analyses because their performance in the experimental task did not differ from their peers. To confirm and justify the inclusion of these individuals, a series of sensitivity analyses were conducted for all the primary measures of interest, which further revealed no significant effect of including versus excluding this group of participants. Therefore, all reported analyses are based on the full sample size of 32 younger and 32 older adults.

### Gap Judgment Accuracy

**FIGURE 4:**
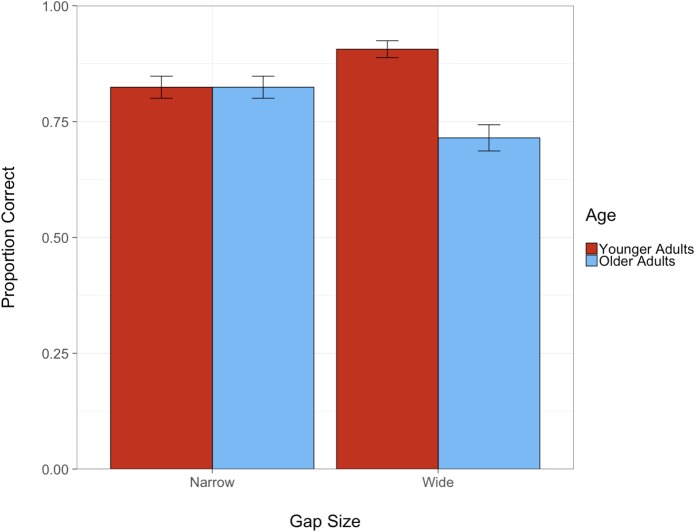
Proportion of correct gap judgments as a function of gap size and age (error bars denote standard error).

The purpose of the gap judgment task was to introduce a need for divided attention during driving in order to strategically evaluate age-related differences in inattentional blindness within a driving context. Thus, in order to ensure that participants were actually performing the task as instructed, and to determine whether there were age-related differences in performing the gap judgment task itself, accuracy scores were calculated and compared between groups. Overall, participants were quite accurate in the gap judgment task (82% overall). In order to determine whether gap judgment accuracy varied as a function of gap size and age, we used a logistic mixed effect model with accuracy as a binary dependent measure (correct vs. incorrect) and gap size (wide vs. narrow), age (younger vs. older), and their corresponding interaction as fixed effects. Whereas there was no effect of gap size on overall accuracy of gap perception judgments, there was an effect of age group with younger adults being more accurate than older adults, β = 0.38, *SE* = 0.19, *Z* = 2.06, *p* = 0.039. This was further qualified by a marginal gap size × age interaction, β = −0.47, *SE* = 0.26, *Z* = −1.82, *p* = 0.069 whereby younger adults were more accurate than older adults in the trials with the wider gap size but not the narrower gap size (Figure 4). Nonetheless, both younger and older adults were overall quite accurate in making the gap judgments (87 and 77%, respectively), suggesting that they were able and compliant in performing the gap judgment task.

### Inattentional Blindness

To measure the rate of inattentional blindness, the analysis file was subsetted to include only the four critical trials in which the forced-choice recognition test was administered. Furthermore, to ensure that participants were engaged in the gap judgment task on each critical trial, all trials in which participants made an incorrect gap judgment were excluded (the average rate of inattentional blindness was no different when incorrect gap judgments were included). The rate of inattentional blindness was measured in terms of accuracy (correct selection of target object during the recognition task), with lower accuracy indicating a higher level of inattentional blindness. The model for the analysis included age, animacy, and their interaction as fixed effects.

**FIGURE 5:**
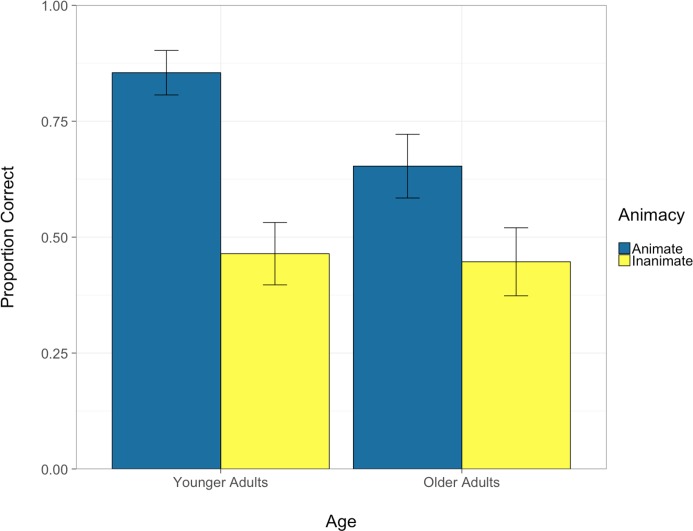
Proportion of correctly identified objects as a function of age and object animacy (error bars denote standard error).

**FIGURE 6:**
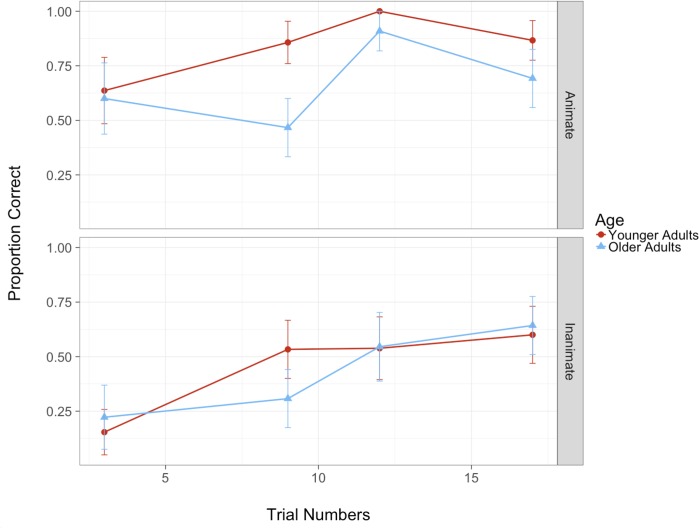
Proportion correct across the four critical trials (3, 9, 12, and 17) as a function of age and animacy (error bars denote standard error).

Results indicated that the only significant effect observed was that of animacy, β = 0.83 *SE* = 0.21, *Z* = 3.99, *p* < 0.001, with better recognition accuracy for animate than inanimate objects for both groups. As illustrated by Figure 5, the differences in detection of animate versus inanimate objects are more pronounced in the younger adults, although this was not statistically significant. We then conducted a follow-up growth curve analysis (Mirman, 2014) to analyze whether inattentional blindness varied across the four critical trials as a function of the order in which they were presented (trial numbers 3, 9, 12, and 17). It is, for instance, possible that after the first critical trial, participants were primed to attend more to environmental objects than they had been previously, which could then have affected their distribution of attentional resources in later trials. The overall time course of accuracy was modeled with a second-order (quadratic) polynomial and included fixed effects of both age and animacy conditions on all time terms. However, due to limited count of observations, the full model would not converge, thus the random effect structure was simplified to include only participant random effects on all time terms. In addition to the effect of animacy, β = 0.95, *SE* = 0.21, *Z* = 4.58, *p* < 0.001, we also observed a significant effect of age group on the linear time term, β = 0.78, *SE* = 0.38, *Z* = 2.04, *p* = 0.042. As illustrated in Figure 6, this is mainly driven by the performance on the second critical trial (Trial 9), whereby younger adults demonstrate reduced inattentional blindness in the second critical trial compared to older adults who did not show this effect. This pattern of results is evident in both animate and inanimate conditions with differences being more pronounced in the former. We describe the implications of this pattern in greater detail in the section “Discussion.”

### Incorrect Recognition Trials

Each response was coded not only in terms of correct and incorrect detection of the critical target, but for the type of incorrect responses, namely whether participants selected the human competitor or a non-human object that never appeared in the scene (e.g., newspaper stand and bicycle). Interestingly, the pattern of results revealed that participants were more likely to pick the non-human object than the human competitor. Furthermore, not only was this pattern of results consistent across both age groups, it was also consistent across trials. In fact, as can be seen in Table 3, it is only the last critical trial in which participants were more likely to incorrectly select the human competitor compared to the non-human object (similar patterns of results are observed after excluding trials with the incorrect gap judgment). We speculate that the reversal of the pattern in the last trial whereby the human competitor was selected more often than the non-human competitor may be due to the overall greater exposure to human characters in the preceding trials.

**Table 3:** The number (percentage) of incorrect decisions for human vs. non-human competitor.

	Younger adults		Older adults	
Trial numbers	Human	Non-human	Human	Non-human
All trials	17 (35%)	31 (65%)	23 (41%)	33 (59%)
3	6 (27%)	16 (73%)	7 (37%)	12 (63%)
9	3 (30%)	7 (70%)	6 (33%)	12 (67%)
12	1 (14%)	6 (86%)	3 (30%)	7 (70%)
17	7 (78%)	2 (22%)	7 (78%)	2 (22%)

## Discussion

In the present study, patterns of inattentional blindness were compared between younger and older adults while they performed a simulated driving task. In particular, we examined whether the awareness of roadside objects differed between the two age groups and whether the animacy of the objects affected awareness. Load was introduced by asking participants to make a perceptual gap judgment about whether they could drive through two rows of parked vehicles. In four critical trials participants were asked to identify roadside objects that differed in terms of their animacy. The results demonstrated that both younger and older adults were significantly more aware of animate compared to inanimate roadside objects, with a trend of this effect being more pronounced in younger compared to older adults. Further, younger adults demonstrated reduced inattentional blindness after the first critical trial, whereas older adults did not show this immediate improvement and continued to exhibit a high rate of inattentional blindness in the second trial. This implies that they did not distribute their attention differentially across the primary driving task and the roadside objects as a function of the prior task demands. Notably, when inattentional blindness *was* observed (i.e., failure to select the correct human), the erroneous choice was significantly more likely to be the non-human object rather than the other human competitor, for both animate and inanimate critical trials and across both age groups, suggesting that they were truly unaware.

### Effects of Age on Inattentional Blindness During Driving

The previously described phenomenon of inattentional blindness during driving in younger adults (Most and Astur, 2007; Blalock et al., 2014; Murphy and Greene, 2015, 2016, 2017a,b; Ericson et al., 2017) was replicated in the current study and was expanded upon by demonstrating the same phenomenon in older adults. Specifically, participants were unaware of inanimate objects on 56% of all trials and animate objects on 25% of all trials (see Table 4). When only considering the very first trial, which is (a) the trial most comparable to other studies of inattentional blindness, which typically test awareness only once, and (b) the only trial preserved against priming or carryover effects, it was observed that participants were unaware of inanimate objects on 82% of the trials and were unaware of animate objects on 38% of the trials. Further, when considering the very last trial, when participants had already been asked three previous times to recognize an object present during driving, performance was still not at ceiling levels with 38% of inattentional blindness observed for inanimate objects and 22% observed for animate objects. This indicates that when performing a moderately difficult driving task, drivers very often lacked conscious awareness of potentially relevant aspects of their surroundings; particularly when they were probed unexpectedly (first trial), but even when they could anticipate being asked (last trial). This observation was bolstered by the fact that recognition errors were almost twice as likely to be due to selecting a non-human object (e.g., bicycle and newspaper stand) rather than the human competitor, suggesting that it was not just a matter of having difficulty distinguishing subtle human features, but rather a general unawareness.

**Table 4:** The rate of inattentional blindness as a function of age and animacy for each trial.

	Younger adults		Older adults	
Trial numbers	Animate	Inanimate	Animate	Inanimate
All trials	16%	54%	33%	57%
3	36%	85%	40%	78%
9	14%	47%	53%	69%
12	0%	46%	9%	45%
17	13%	40%	31%	36%

Interestingly, however, older adults did not demonstrate overall higher or lower rates of inattentional blindness compared to younger adults, counter to initial predictions. This suggests that, within the constraints of this task, there was no evidence to support older adults’ *reduced* awareness of roadside objects due to a generally *lower* attentional capacity, or an *increased* awareness of roadside objects due to *poorer* inhibitory control. There was, however, some indication that older adults did not as rapidly adjust their distribution of attentional resources after learning from previous trial demands. Specifically, whereas younger adults demonstrated significantly reduced inattentional blindness after having already been previously prompted to attend to roadside objects by the recognition task (perhaps priming them to anticipate that they may have to divide their attention in order to recognize future roadside objects), older adults did not. The need for flexible adjustment of task demands could be particularly important in the context of real world driving.

The largely comparable levels of inattentional blindness in younger and older adults observed in this study are different from some prior studies demonstrating higher rates of inattentional blindness in older compared to younger adults in non-driving tasks (e.g., Graham and Burke, 2011). The current results are also different from some driving-context specific studies of hazard detection that have shown lower detection rates in older compared to younger drivers (e.g., Bromberg et al., 2012; Feng et al., 2018; although see Borowsky et al., 2010). However, the results of the current study *are* consistent with other previous studies reporting measures of awareness as evidenced through explicit detection tasks and actual driving performance metrics under conditions of load (e.g., Strayer and Drews, 2004; Stinchcombe and Gagnon, 2013). For instance, Stinchcombe and Gagnon (2013) reported no age-related differences between middle aged and older drivers for driving performance measures or peripheral detection task accuracy during complex driving tasks known to be associated with real world collisions. Further, Strayer and Drews (2004) reported that while both older and younger adults were negatively affected by talking on a cell phone during simulated driving (e.g., slower reaction times and increased rear-end collisions), there were no age-related differences. Taken together, in the below discussion we consider the parameters that differ across these studies to highlight the potential role that particular factors may play in the observed results including, the nature of the task (e.g., recall vs. recognition vs. driving performance), the magnitude of load (e.g., lower vs. moderate vs. higher perceptual/cognitive load), the nature of the “unexpected” object/feature (e.g., relevance to the primary task), and how these factors may interact with age.

One of the primary differences across studies of inattentional blindness, situational awareness, and hazard detection across driving and non-driving tasks relates to the way that “awareness” is operationalized and measured. For instance, in the current study, a post-drive recognition task was used, whereas other studies of inattentional blindness have asked participants to freely recall an object/event (e.g., “did you notice anything different/unusual on the last trial,” Graham and Burke, 2011; Murphy and Greene, 2016), and yet others have considered driving performance measures like brake reaction times (e.g., Strayer and Drews, 2004; Ericson et al., 2017). These different task types may be uniquely targeting implicit versus explicit levels of awareness. Therefore, it is possible that older adults may have a reduced conscious awareness of scene/object differences across trials compared to younger adults (e.g., poorer recall accuracy; Graham and Burke, 2011), but they may still have an implicit awareness of having seen that object with comparable accuracy to younger adults (i.e., similar recognition accuracy as was observed in the current study). Indeed, there is significant evidence in the general aging and cognition literature that recall is more significantly affected by older age than is recognition (Craik and McDowd, 1987; Danckert and Craik, 2013) and explicit memory is more significantly affected (or oppositely affected) by older age than implicit memory (La Voie and Light, 1994; Gopie et al., 2011). There are also interesting implications regarding these distinctions when considering how implicit and explicit awareness are associated with actual driving performance measures. It is likely that even without explicit or conscious awareness, implicit detection may result in associated changes in driving performance. This interpretation is consistent with the agreement between comparable performance by older and younger drivers on the recognition-based responses in the current study and the comparable performance of older and younger drivers reported for other driving performance related measures across other studies (e.g., Strayer and Drews, 2004).

Differences across studies could also relate to the various types and levels of perceptual and/or cognitive load that are introduced (Lavie, 2005; Murphy et al., 2016) and the different effects of load on older compared to younger adults. It may be that under low load conditions younger and older drivers perform similarly well and under high load conditions younger and older drivers perform similarly poorly. Therefore, it may be during moderately loaded conditions that age-related effects are best revealed. Given that it is difficult to normalize load across studies, it is possible that the load introduced in the current study was higher or lower than in other previous studies of age-related effects on inattentional blindness, and/or age-related effects of object awareness during driving. It is also possible that the different methods of stimulus presentation could be contributing to differences in age-related effects. For instance, smaller field-of-view displays and/or video or photo-based stimuli may result in different age-related effects compared to larger field-of-view displays, or immersive simulation systems, as well as where the stimuli appear within these displays (e.g., within or outside of the useful field of view).

### Effects of Animacy on Inattentional Blindness During Driving

In addition to the effects of the context, task, and load on inattentional blindness, the relevance of the unexpected object to the primary task itself may also be a contributing factor. In inattentional blindness studies, the target object of interest is often referred to as “irrelevant” to the main task or “unexpected.” The same may not be true in contextualized tasks such as driving where the environmental features and objects can differ and vary dynamically in terms of their relevance (e.g., proximity to roadway, or ability to interfere with the primary driving task) (Pammer and Blink, 2013; Pammer et al., 2015; Topolšek et al., 2016; Murphy and Greene, 2017a). Animacy is a characteristic that is particularly relevant in the context of driving given that it introduces the increased probability that the object could interfere/interact with the driving task. The results of the current study revealed a highly significant effect of animacy, with much higher recognition rates of animate compared to inanimate objects. Importantly, the physical features of the animate and inanimate objects in this study were essentially identical, with the difference being how the object was contextualized (i.e., embedded in an advertisement or not). This effect of animacy was also observed across age groups and across trials and the effect is consistent with past studies involving both non-driving tasks as well as driving relevant scenes (Pammer and Blink, 2013; Calvillo and Jackson, 2014; Pammer et al., 2015; Calvillo and Hawkins, 2016; Topolšek et al., 2016).

Even though there was no significant interaction effect between age group and animacy, it is interesting to note that younger adults demonstrated quite a high rate of awareness for animate objects across trials (84%) compared to older adults who were relatively poorer (67%); which was in contrast to the inanimate trials, for which younger and older adults were much more comparable to each other (46 and 43%, respectively). This suggests that the influence of animacy on response selection was more pronounced for younger adults than older adults. The implications for this during a real driving context could mean that older adults may not be as strategically attending to potentially relevant environmental information in the same way as younger adults (Bromberg et al., 2012; Horwood and Beanland, 2016; Feng et al., 2018).

### Potential Limitations and Future Directions

Although the current study was targeted at evaluating age-related effects on inattentional blindness during driving, it did not control for between-group differences in terms of lifetime history of driving experience. Whereas it was ensured that all participants had valid driver’s licenses and that there were no statistically significant between-group differences in current driving habits (i.e., average km driven per week), older adults likely had driven for more years total than younger adults. Therefore, greater experience with driving overall may have allowed older adults to use acquired driving skills to compensate for any potential age-related declines in sensory, motor, or cognitive abilities, resulting in no overall age-related differences on task performance. Yet another possibility is that even though all participants received the same instructions to drive constantly at 80 km/h, with compliance verified during the practice trials, perhaps during experimental trials older adults modulated their speed differently than younger adults. To explore this possibility, we verified the average speed of participants across the four critical trials (from the onset of the sound to the first parked vehicle). We found that younger and older adults were able to maintain the target speed with good accuracy (*M*_Older_ = 79 km/h and *M*_Y ounger_ = 84 km/h). Nonetheless, it is quite possible that when a speed limit is not strictly enforced, older adults may slow down in order to better manage the multiple tasks of driving (gap judgment and driving maneuvers), while also remaining aware of their surroundings (recognition task performance) (Bromberg et al., 2012). Similarly, even though all participants were asked to drive through the gap when it was wide enough to clear, older adults may have taken a more conservative approach and opted to drive around the vehicles during larger gap sizes than younger adults, even if they perceived it to be wide enough to fit through.

**FIGURE 7:**
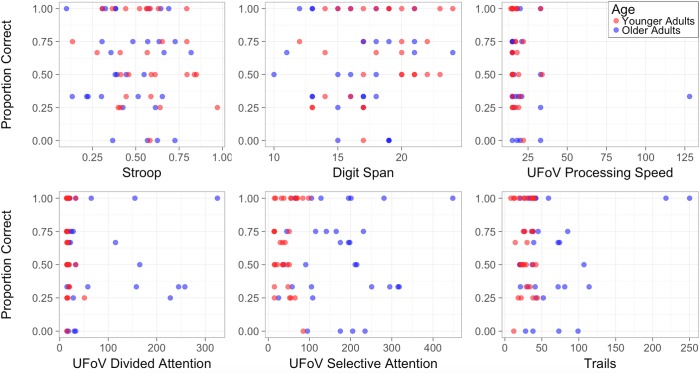
Relation between cognitive assessments and the experimental recognition task performance (Stroop: number of correct words per second from neutral to incongruent condition; Digit Span: correct scores out of 30; UFoV measures are calculated in milliseconds; Trails: the difference in seconds between Trail B-A).

The older adult sample included here may also not be representative of the wider older adult driving population due to the strict eligibility criteria requiring no sensory, motor, or cognitive impairments and requiring an active driving status (licensed and frequent drivers). Indeed, the younger and older adult groups in this study were generally well matched on baseline tests of general functioning. For example, there were no significant between-group differences on tests of working memory (digit span), inhibition (Stroop), and processing speed (UFoV subset). Likewise, there were no observable differences in terms of participants who scored below the cutoff for mild cognitive impairment on the MoCA (see also Rapoport et al., 2013). However, older adults did perform significantly poorer on the Trail making test (visual attention and task switching) and the divided and selective attention subsets of the UFoV test. In order to explore the potential associations between individual participant’s scores on the baseline measures and their recognition task performance during the main driving experiment, these data were plotted relative to each other (Figure 7). Visual inspection suggests that, for older adults, faster performance on the UFoV divided and selective attention tasks may be associated with better recognition task performance during driving. However, because of the nature of the binary recognition task measure, analyses to test for statistical associations were not possible. It is, therefore, evident that more studies are required to determine the role of individual differences on inattentional blindness in younger and older adults, particularly in the context of driving. Moreover, although the current sample size was similar to previous studies comparing younger and older drivers (e.g., Strayer and Drews, 2004; Stinchcombe and Gagnon, 2013), it may have lacked the sufficient power to detect more subtle age-related differences.

Because the same characters were presented in the same trial order either as animate or inanimate, there might be concern that the characteristic of the particular object in that trial could have differentially influenced the performance, despite the efforts made to ensure that characters were similar in composition, size, and saliency. In order to examine whether target features could have differentially affected recognition, we compared performance differences across different target types and observed no discernable patterns. For instance, we considered whether the sex of the target person affected recognition performance, but it did not appear to, given that the low performance observed in the second critical trial was for a female target and highest performance in the third critical trial was also a female target. Similarly, the first trial with low performance was a male in a suit, but the last probe trial with perfect performance was also a male in a suit.

Finally, as is the case for all simulator studies, the effects observed here may not generalize completely to real world driving. Particularly relevant here is that the consequence of making a gap judgment error (e.g., driving through a gap that is too small) in the simulator is much more benign than if the same error were made during real on-road driving. This consideration may also be influenced by age, as the consequences of a collision for older adults is likely to be much more serious than for younger adults given increases in fragility with age and poorer outcomes associated with injury and recovery (Vichitvanichphong et al., 2015).

## Conclusion

Overall, the current results demonstrate that younger and older drivers had similar rates of inattentional blindness when evaluated using a recognition task within a driving paradigm. The most robust factor affecting inattentional blindness was the animacy of the roadside object, with animate objects being recognized significantly more often than inanimate objects. The effects of inattentional blindness were most pronounced on the very first trial, but persisted even after being primed three times prior. While younger adults appeared to distribute their attention more strategically after becoming aware of the potential task of recognizing roadside objects after the first trial, it took more trials for the older adults to redistribute their attention. Factors associated with whether age-related changes influence the rate of inattentional blindness could include the nature of the task/context, the magnitude of perceptual and cognitive load, and the features of the environment to be attended and/or ignored.

## Ethics Statement

All subjects gave written informed consent in accordance with the Declaration of Helsinki. The protocol was approved by the University Health Network’s Research Ethics Board (REB 17-5596).

## Author Contributions

RS and JC conceived the study. RS and KB collected the data. RS, JC, and KB contributed to the analyses and to the writing of the manuscript.

## Conflict of Interest Statement

The authors declare that the research was conducted in the absence of any commercial or financial relationships that could be construed as a potential conflict of interest.

## References

[B1] Anderson J. A. Campbell K. L. Amer T. Grady C. L. Hasher L. (2014). Timing is everything: age differences in the cognitive control network are modulated by time of day. *Psychol. Aging* 29 648–657. 10.1037/a0037243 24999661 PMC4898963

[B2] Ball K. Owsley C. (1993). The useful field of view test: a new technique for evaluating age-related declines in visual function. *J. Am. Optom. Assoc.* 64 71–79. 8454831

[B3] Bates D. Maechler B. Bolker B. Walker S. (2015). Fitting linear mixed-effects models using lme4. *J. Stat. Softw.* 67 1–48. 10.18637/jss.v067.i01

[B4] Blalock L. D. Sawyer B. D. Kiken A. Gutzwiller R. S. McGill C. L. Clegg B. A. (2014). Cognitive load while driving impairs memory of moving but not stationary elements within the environment. *J. Appl. Res. Mem. Cogn.* 3 95–100. 10.1016/j.jarmac.2014.04.006

[B5] Borowsky A. Shinar D. Oron-Gilad T. (2010). Age, skill, and hazard perception in driving. *Accid. Anal. Prev.* 42 1240–1249. 10.1016/j.aap.2010.02.001 20441838

[B6] Bromberg S. Oron-Gilad T. Ronen A. Borowsky A. Parmet Y. (2012). The perception of pedestrians from the perspective of elderly experienced and experienced drivers. *Accid. Anal. Prev.* 44 48–55. 10.1016/j.aap.2010.12.028 22062336

[B7] Caird J. K. Simmons S. M. Wiley K. Johnston K. A. Horrey W. J. (2018). Does talking on a cell phone, with a passenger, or dialing affect driving performance? An updated systematic review and meta-analysis of experimental studies. *Hum. Factors* 60 101–133. 10.1177/0018720817748145 29351023

[B8] Calvillo D. P. Hawkins W. C. (2016). Animate objects are detected more frequently than inanimate objects in inattentional blindness tasks independently of threat. *J. Gen. Psychol.* 143 101–115. 10.1080/00221309.2016.1163249 27055078

[B9] Calvillo D. P. Jackson R. E. (2014). Animacy, perceptual load, and inattentional blindness. *Psychon. Bull. Rev.* 21 670–675. 10.3758/s13423-013-0543-8 24197657

[B10] Cantin V. Lavallière M. Simoneau M. Teasdale N. (2009). Mental workload when driving in a simulator: effects of age and driving complexity. *Accid. Anal. Prev.* 41 763–771. 10.1016/j.aap.2009.03.019 19540965

[B11] Craik F. I. McDowd J. M. (1987). Age differences in recall and recognition. *J. Exp. Psychol. Learn. Mem. Cogn.* 13 474–479. 10.1037/0278-7393.13.3.474

[B12] Cuenen A. Jongen E. M. Brijs T. Brijs K. Lutin M. Van Vlierden K. (2015). Does attention capacity moderate the effect of driver distraction in older drivers? *Accid. Anal. Prev.* 77 12–20. 10.1016/j.aap.2015.01.011 25667202

[B13] Danckert S. L. Craik F. I. (2013). Does aging affect recall more than recognition memory? *Psychol. Aging* 28 902–909. 10.1037/a0033263 23978011

[B14] Donmez B. Liu Z. (2015). Associations of distraction involvement and age with driver injury severities. *J. Saf. Res.* 52 23–28. 10.1016/j.jsr.2014.12.001 25662879

[B15] Ebnali M. Ahmadnezhad P. Shateri A. Mazloumi A. Heidari M. E. Nazeri A. R. (2016). The effects of cognitively demanding dual-task driving condition on elderly people’s driving performance, Real driving monitoring. *Accid. Anal. Prev.* 94 198–206. 10.1016/j.aap.2016.05.016 27328019

[B16] Ericson J. M. Parr S. A. Beck M. R. Wolshon B. (2017). Compensating for failed attention while driving. *Transp. Res. F Traffic Psychol. Behav.* 45 65–74. 10.1016/j.trf.2016.11.015

[B17] Feng J. Choi H. Craik F. I. Levine B. Moreno S. Naglie G. (2018). Adaptive response criteria in road hazard detection among older drivers. *Traffic Inj. Prev.* 19 141–146. 10.1080/15389588.2017.1373190 28898116 PMC5921861

[B18] Ferris F. L. III Kassoff A. Bresnick G. H. Bailey I. (1982). New visual acuity charts for clinical research. *Am. J. Ophthalmol.* 94 91–96. 10.1016/0002-9394(82)90197-0 7091289

[B19] Golding J. F. (2006). Predicting individual differences in motion sickness susceptibility by questionnaire. *Pers. Individ. Dif.* 41 237–248. 10.1016/j.paid.2006.01.012 12132644

[B20] Gopie N. Craik F. I. Hasher L. (2011). A double dissociation of implicit and explicit memory in younger and older adults. *Psychol. Sci.* 22 634–640. 10.1177/0956797611403321 21421935

[B21] Graham E. R. Burke D. M. (2011). Aging increases inattentional blindness to the gorilla in our midst. *Psychol. Aging* 26 162–166. 10.1037/a0020647 21261412 PMC3062668

[B22] Hasher L. Zacks R. T. (1988). “Working memory, comprehension, and aging: a review and a new view,” in *Psychology of Learning and Motivation* Vol. 22 ed. Bower G. H., (New York, NY: Academic Press), 193–225.

[B23] Horrey W. J. Wickens C. D. (2006). Examining the impact of cell phone conversations on driving using meta-analytic techniques. *Hum. Factors* 48 196–205. 10.1518/001872006776412135 16696268

[B24] Horwood S. Beanland V. (2016). Inattentional blindness in older adults: effects of attentional set and to-be-ignored distractors. *Attent. Percept. Psychophys.* 78 818–828. 10.3758/s13414-015-1057-4 26758974

[B25] International Council of Ophthalmology (2002). *Visual* *Standards Aspects and Ranges of Vision Loss with Emphasis on Population Surveys* Available at: www.icoph.org/pdf/visualstandardsreport.pdf (accessed November 3, 2018).

[B26] Jackson L. Cracknell R. (2018). *Road Accident Casualties in Britain and the World.* Available at: https://researchbriefings.parliament.uk/ResearchBriefing/Summary/CBP-7615 (accessed April 23, 2018).

[B27] Kass S. J. Cole K. S. Stanny C. J. (2007). Effects of distraction and experience on situation awareness and simulated driving. *Transp. Res. F Traffic Psychol. Behav.* 10 321–329. 10.1016/j.trf.2006.12.002 16310750

[B28] Keshavarz B. Hecht H. (2011). Validating an efficient method to quantify motion sickness. *Hum. Factors* 53 415–426. 10.1177/0018720811403736 21901938

[B29] Kreitz C. Furley P. Memmert D. Simons D. J. (2016). The influence of attention set, working memory capacity, and expectations on inattentional blindness. *Perception* 45 386–399. 10.1177/0301006615614465 26562879

[B30] Kuznetsova A. Brockhoff P. B. Christensen R. H. B. (2017). lmerTest package: tests in linear mixed effects models. *J. Stat. Softw.* 82 1–26. 10.18637/jss.v082.i13

[B31] La Voie D. Light L. L. (1994). Adult age differences in repetition priming: a meta-analysis. *Psychol. Aging* 9 539–553. 10.1037/0882-7974.9.4.539 7893425

[B32] Lavie N. (2005). Distracted and confused?: Selective attention under load. *Trends Cogn. Sci.* 9 75–82. 10.1016/j.tics.2004.12.004 15668100

[B33] Lavie N. Ro T. Russell C. (2003). The role of perceptual load in processing distractor faces. *Psychol. Sci.* 14 510–515. 10.1111/1467-9280.03453 12930485

[B34] Liu H. (2018). Age-related effects of stimulus type and congruency on inattentional blindness. *Front. Psychol.* 9:794. 10.3389/fpsyg.2018.00794 29875724 PMC5974594

[B35] Lustig C. Hasher L. Zacks R. T. (2007). Inhibitory deficit theory: recent developments in a “new view”. *Inhib. Cogn.* 17 145–162. 10.1037/11587-008

[B36] Mack A. Rock I. (1998). *Inattentional Blindness.* Cambridge, MA: MIT Press 10.7551/mitpress/3707.001.0001

[B37] Marciano H. Yeshurun Y. (2012). Perceptual load in central and peripheral regions and its effects on driving performance: advertizing billboards. *Work* 41 3181–3188. 10.3233/WOR-2012-0580-3181 22317201

[B38] Marciano H. Yeshurun Y. (2015). Perceptual load in different regions of the visual scene and its Relevance for driving. *Hum. Factors* 54 701–716. 10.1177/0018720814556309 25977327

[B39] McDowd J. M. Craik F. I. (1988). Effects of aging and task difficulty on divided attention performance. *J. Exp. Psychol. Hum. Percept. Perform.* 14 267–280. 10.1037/0096-1523.14.2.267 2967880

[B40] Michaels J. Chaumillon R. Nguyen-Tri D. Watanabe D. Hirsch P. Bellavance F. (2017). Driving simulator scenarios and measures to faithfully evaluate risky driving behavior: a comparative study of different driver age groups. *PLoS One* 12:e0185909. 10.1371/journal.pone.0185909 29016693 PMC5634611

[B41] Mirman. (2014). *Growth Curve Analysis and Visualization Using R.* Boca Raton, FL: Chapman and Hall.

[B42] Most S. B. Astur R. S. (2007). Feature-based attentional set as a cause of traffic accidents. *Vis. Cogn.* 15 125–132. 10.1080/13506280600959316

[B43] Murphy G. Greene C. M. (2015). High perceptual load causes inattentional blindness and deafness in drivers. *Vis. Cogn.* 23 810–814. 10.1080/13506285.2015.1093245

[B44] Murphy G. Greene C. M. (2016). Perceptual load induces inattentional blindness in drivers. *Appl. Cogn. Psychol.* 30 479–483. 10.1002/acp.3216

[B45] Murphy G. Greene C. M. (2017a). Load theory behind the wheel; perceptual and cognitive load effects. *Can. J. Exp. Psychol.* 71 191–202. 10.1037/cep0000107 28604027

[B46] Murphy G. Greene C. M. (2017b). The elephant in the road: auditory perceptual load affects driver perception and awareness. *Appl. Cogn. Psychol.* 31 258–263. 10.1002/acp.3311

[B47] Murphy G. Groeger J. A. Greene C. M. (2016). Twenty years of load theory — Where are we now, and where should we go next? *Psychon. Bull. Rev.* 23 1316–1340. 10.3758/s13423-015-0982-5 26728138

[B48] Nasreddine Z. S. Phillips N. A. Bédirian V. Charbonneau S. Whitehead V. Collin I. (2005). The Montreal Cognitive Assessment, MoCA: a brief screening tool for mild cognitive impairment. *J. Am. Geriatr. Soc.* 53 695–699. 10.1111/j.1532-5415.2005.53221.x 15817019

[B49] Owsley C. Stalvey B. Wells J. Sloane M. E. (1999). Older drivers and cataract: driving habits and crash risk. *J. Gerontol. Med. Sci.* 54A, M203–M211. 10.1093/gerona/54.4.m203 10219012

[B50] Pammer K. Bairnsfather J. Burns J. Hellsing A. (2015). Not all hazards are created equal: the significance of hazards in inattentional blindness for static driving scenes. *Appl. Cogn. Psychol.* 29 782–788. 10.1002/acp.3153

[B51] Pammer K. Blink C. (2013). Attentional differences in driving judgments for country and city scenes: semantic congruency in inattentional blindness. *Accid. Anal. Prev.* 50 955–963. 10.1016/j.aap.2012.07.026 22975367

[B52] R Core Team (2017). *R: A Language and Environment for Statistical Computing.* Vienna: R Foundation for Statistical Computing.

[B53] Rapoport M. J. Naglie G. Weegar K. Myers A. Cameron D. Crizzle A. (2013). The relationship between cognitive performance, perceptions of driving comfort and abilities, and self-reported driving restrictions among healthy older drivers. *Accid. Anal. Prev.* 61 288–295. 10.1016/j.aap.2013.03.030 23601097

[B54] Reitan R. M. (1955). The relation of the Trail Making Test to organic brain damage. *J. Consult. Psychol.* 19 393–394. 10.1037/h0044509 13263471

[B55] Road Safety Canada (2011). *Road Safety in Canada Report.* Ottawa: The Public Health Agency of Canada.

[B56] Shinar D. Tractinsky N. Compton R. (2005). Effects of practice, age, and task demands, on interference from a phone task while driving. *Accid. Anal. Prev.* 37 315–326. 10.1016/j.aap.2004.09.007 15667818

[B57] Simons D. J. Chabris C. F. (1999). Gorillas in our midst: sustained inattentional blindness for dynamic events. *Perception* 28 1059–1074. 10.1068/p281059 10694957

[B58] Smahel T. Smiley A. Donderi D. (2008). “The effects of cellular phone use on novice and experienced driver performance: an on-road study,” in *Proceedings of the Human Factors and Ergonomics Society Annual Meeting*, Vol. 52 (Thousand Oaks, CA: Sage Publications), 1910–1914. 10.1177/154193120805202317

[B59] Stinchcombe A. Gagnon S. (2013). Aging and driving in a complex world: exploring age differences in attentional demand while driving. *Transp. Res. F Traffic Psychol. Behav.* 17 125–133. 10.1016/j.trf.2012.11.002

[B60] Stothart C. Boot W. Simons D. (2015). Using Mechanical Turk to assess the effects of age and spatial proximity on inattentional blindness. *Collabra* 1 1–7. 10.1525/collabra.26

[B61] Stothart C. Boot W. R. Simons D. Charness N. Wright T. (2016). “Age effects on inattentional blindness: implications for driving,” in *Human Aspects of IT for the Aged Population. Healthy and Active Aging*, eds Zhou J. Salvendy G. (Cham: Springer), 10.1007/978-3-319-39949-2_42

[B62] Strayer D. L. Cooper J. M. Turrill J. Coleman J. Medeiros-Ward N. Biondi F. (2013). *Measuring Cognitive Distraction in the Automobile.* Washington, DC: AAA Foundation for Traffic Safety.

[B63] Strayer D. L. Drews F. A. (2004). Profiles in driver distraction: effects of cell phone conversations on younger and older drivers. *Hum. Factors* 46 640–649. 10.1518/hfes.46.4.640.56806 15709326

[B64] Strayer D. L. Drews F. A. Johnston W. A. (2003). Cell phone-induced failures of visual attention during simulated driving. *J. Exp. Psychol. Appl.* 9 23–32. 10.1037/1076-898x.9.1.23 12710835

[B65] Strayer D. L. Johnston W. A. (2001). Driven to distraction: dual-task studies of simulated driving and conversing on a cellular telephone. *Psychol. Sci.* 12 462–466. 10.1111/1467-9280.00386 11760132

[B66] Stroop J. R. (1935). Studies of interference in serial verbal reactions. *J. Exp. Psychol.* 18 643–662. 10.1037/h0054651

[B67] Svetina M. (2016). The reaction times of drivers aged 20 to 80 during a divided attention driving. *Traffic Inj. Prev.* 17 810–814. 10.1080/15389588.2016.1157590 26980290

[B68] The MathWorks Inc. (2015). *MATLAB and Statistics Toolbox Release 2015b.* Natick, MA: United States.

[B69] Topolšek D. Areh I. Cvahte T. (2016). Examination of driver detection of roadside traffic signs and advertisements using eye tracking. *Transp. Res. F Traffic Psychol. Behav.* 43 212–224. 10.1016/j.trf.2016.10.002

[B70] Transport Canada (2014). *Canadian Motor Vehicle Traffic Collision Statistics.* Available at: https://www.tc.gc.ca/eng/motorvehiclesafety/resources-researchstats-menu-847.htm (accessed May 22, 2017).

[B71] Vichitvanichphong S. Talaei-Khoei A. Kerr D. Ghapanchi A. H. (2015). What does happen to our driving when we get older? *Transp. Rev.* 35 56–81. 10.1080/01441647.2014.997819

[B72] Wechsler D. (1997). *Wechsler Adult Intelligence Scale*, 3rd Edn. San Antonio, TX: The Psychological Corporation.

[B73] Wechsler K. Drescher U. Janouch C. Haeger M. Voelcker-Rehage C. Bock O. (2018). Multitasking during simulated car driving: a comparison of young and older persons. *Front. Psychol.* 9:910. 10.3389/fpsyg.2018.00910 29962983 PMC6013591

